# Indirect immunofluorescent assay as an aid in the diagnosis of suspected immune mediated ataxias

**DOI:** 10.1186/s40673-021-00129-1

**Published:** 2021-02-16

**Authors:** Marios Hadjivassiliou, Graeme Wild, Priya Shanmugarajah, Richard A. Grünewald, Mohammed Akil

**Affiliations:** 1 Academic Department of Neurosciences, Sheffield Teaching Hospitals NHS Trust and University of Sheffield, Royal Hallamshire Hospital, Glossop Road, Sheffield, S10 2JF UK; 2 Clinical Immunology and Allergy Unit, Sheffield Teaching Hospitals NHS Trust, Sheffield, UK; 3 Rheumatology Department, Sheffield Teaching Hospitals NHS Trust, Sheffield, UK

**Keywords:** Immune mediated cerebellar ataxia, Immunofluorescence, Primary autoimmune cerebellar ataxia

## Abstract

**Background and purpose:**

Immune mediated cerebellar ataxias account for a substantial proportion of all progressive ataxias. A diagnostic serological test is not always available. This is particularly problematic in Primary Autoimmune Cerebellar Ataxia, hence the necessity for diagnostic criteria recently devised and published by an International Task Force. We present our experience in the use of a commercially available indirect immunofluorescence assay, intended to be used for the detection of antibodies associated with paraneoplastic neurological syndromes.

**Methods:**

Retrospective review of patients with ataxia who underwent serological testing using this assay as part of their diagnostic evaluation. We were interested in 3 groups: suspected immune mediated ataxias, genetically confirmed ataxias and patients with cerebellar variant of multi-system atrophy (MSA-C). The indirect immunofluorescence assay was performed using commercially available monkey cerebellum slides and anti-human IgG FITC conjugated antiserum.

**Results:**

A total of 300 patients that had this test and fitted into one of these 3 groups (immune ataxias 190, genetic ataxias 60, MSA-C 50) were identified. The prevalence of positive immunofluorescence but negative immunoblot was 172/190 (91%) in the suspected immune ataxia group, 3/60 (5%) in the genetic group and 2/50 (4%) in the MSA-C group. The difference between the first and the other groups was significant *χ*^*2*^ (1, *N* = 291) = 64.2, *p* < 00001.

**Conclusions:**

This report demonstrates that a commercially available immunofluorescence assay can be used to provide additional diagnostic aid for suspected immune mediated ataxias and in particular Primary Autoimmune Cerebellar Ataxia where no diagnostic marker exists.

## Introduction

Immune mediated cerebellar ataxias (IMCA) account for a substantial proportion of all progressive ataxias [1]. Examples of IMCA include Gluten Ataxia (GA), anti-GAD ataxia, Paraneoplastic Cerebellar Degeneration (PCD) and post-infectious cerebellitis. Unlike GA, PCD and post-infectious cerebellitis, in which an antigenic trigger is known, in some suspected autoimmune ataxias the antigenic trigger is not known and any associated neuronal antibodies are not well characterized or of proven pathogenicity. The term Primary Autoimmune Cerebellar Ataxia (PACA) was introduced to describe this latter group of patients [2].

It is very likely that a substantial number of patients with late onset so called “idiopathic” sporadic ataxias have PACA [1]. Recently an International Task Force on IMCA published diagnostic criteria for PACA in order to enable neurologists to consider this diagnosis [3]. The proposed diagnostic criteria for PACA are based on clinical (mode of onset, pattern of cerebellar involvement, presence of other autoimmune diseases), imaging findings (MRI and if available MR spectroscopy showing preferential, but not exclusive involvement of vermis) and laboratory investigations (CSF pleocytosis and/or CSF-restricted IgG oligoclonal bands) parameters. These criteria are important because PACA is potentially treatable with immunosuppression [4].

Aside of the clinical criteria for the diagnosis of PACA, investigations such as CSF examination (pleocytosis and/or oligoclonal bands) and serological evidence of antibodies associated with additional autoimmune diseases (which can be a feature in PACA) can also be useful clues to its diagnosis. However, so far there is no single investigation that can be viewed as a reliable diagnostic marker for PACA.

Here, we describe our observations and experience in the use of a commercially available indirect immunofluorescence assay using monkey cerebellum, originally intended to be used for the detection of paraneoplastic antibodies in the context of paraneoplastic neurological syndromes including PCD.

## Methods

### Patient selection

All patients reported here were attending the Sheffield Ataxia Centre. They underwent a battery of investigations as part of their diagnostic workup for causes of ataxia. Details of all these tests can be found in a previous publication from our group [1]. Genetic testing was performed with next generation sequencing using a panel of 175 ataxia genes (for details of the genes see supplementary material).

The patient selection for this report was retrospective. The selection was based on all patients with ataxia who had undergone paraneoplastic antibody screening as part of their ataxia diagnostic work up. All patients had completed their clinical evaluation and investigations (blood tests, genetic tests and brain imaging) and had been (and still are) followed up at regular intervals. We selected all the patients that fell into 3 of the following groups:

1. Patients with IMCA (gluten ataxia, anti-GAD ataxia, or fulfilling the criteria for PACA). Those patients already on immunosuppression or on gluten-free diet at the time of the baseline testing were excluded. We also excluded patients with PCD. Patients with gluten ataxia by definition were positive for antigliadin antibodies. Patients with anti-GAD ataxia were positive for anti-GAD antibodies (titre > 2000). Patients with PACA fulfilled the published criteria for the diagnosis of PACA [3]. None of the patients in this IMCA group were found to have an alternative explanation for their ataxia. Detail clinical characterisation of these 3 different types of immune ataxias have already been published [4–6].
2. Patients with genetically confirmed ataxia.
3. Patients with clinically probable cerebellar variant of multiple system atrophy (MSA-C).

The rational for selecting patients in groups 2 and 3 was for a suitable control groups of ataxia patients with different pathogenesis (genetic or degenerative respectively).

### Indirect immunofluorescence

The indirect immunofluorescence assay was performed using commercially available monkey cerebellum slides and anti-human IgG FITC conjugated antiserum (Inova Diagnostics). Wells on the slides were flooded with 1/50 diluted samples in PBS. The slides were incubated for 30 min and then washed with PBS before flooding with anti-human IgG FITC conjugated antiserum. Slides were incubated for 30 min, washed again in PBS and then examined under a UV microscope at 470 nm. Cerebellar auto-antibodies can be identified by their characteristic fluorescent staining patterns: Yo - Cytoplasm of Purkinje Cells, Hu - Cell nuclei of neurons in the CNS and PNS, Amphiphysin - Membrane of synaptic vesicles, CV2 - Cytoplasm of Oligodendrocytes, Ri - Cell nuclei of neurons in the CNS, Tr - Cytoplasm of Purkinje cells and dots in the molecular layer.

Positive immunofluorescent patterns were confirmed by EUROLINE immunoblots (Euroimmun AG) on a EUROBlotOne (Euroimmun AG) automated processor. The EUROLINE immunoblot contains antigens for Amphiphysin, CV2.1, PNMA2 (Ma2/Ta), Ri, Yo, and Hu. Bands on the individual immunoblots are qualitatively reported according to their intensity. We interpret the readings as 0 (negative) + (negative) and ++ (weak positive) and +++ (positive). For the purpose of this report the patients were divided into positive (++, +++) or negative (no staining, +).

Positive fluorescence results on any sample with a negative immunoblot were reported as per the pattern seen (Yo-like, Hu-like etc).

## Results

A total of 300 patients who underwent serological testing for paraneoplastic antibodies using the above assay are included in this report. Of these, 190 were considered as having IMCA. This group included patients with gluten ataxia (143), patients fulfilling the criteria for PACA (32) and patients with anti-GAD ataxia (15). Group 2 consisted of 60 patients who had a confirmed genetic diagnosis. Group 3 consisted of 50 patients with clinically probable MSA-C. The prevalence of positive immunofluorescence but negative immunoblot was 172/190 (91%) in the suspected IMCA group, 3/60 (5%) in the genetic group and 2/50 (4%) in the MSA-C group. The difference between the first group and the other 2 groups was significant *χ*^*2*^ (1, *N* = 291) = 64.2, *p* < 00001. By far the commonest pattern of staining in the positive cases was Hu-like (141) followed by Yo-like (11). Much less common patterns included CV2-like, Ma2-like, amphyphysin-like and Tr-like. Table 1 summarises the results.

**Table 1:** summary of the immunofluorescence findings per group of patients with immune, genetic or degenerative ataxias

	Immune mediated cerebellar ataxias (190 patients)			Control groups of ataxias (110 patients)	
Number of patients positive on immunofluorescence (percentage positive)	172/190 (91%)			5/110 (5%)	
Ataxia subgroups (number of patients)	Gluten Ataxia (143)	Primary Autoimmune Carebellar Ataxias (32)	Anti-GAD Ataxia (15)	Genetically Confirmed Ataxias (60)	Cerebellar variant of Multi-System Atrophy (50)
Number of patients with positive immunofluorescence per group (percentage positive)	126/143 (88%)	32/32 (100%)	14/15 (93%)	3/60 (5%)	2/40 (4%)
Type of staining per group (percentage from positive patients)	Hu like 109/126 (87%) Yo like 7/126 (6%) CV2 like 4/126 (3%) amphiphysin like 3/126 (2%) Tr like 2/126 (2%) Ma2 like 1/126 (1%)	Hu like 26/32 (81%) Yo like 4/32 (13%) (one had both Hu and Yo like staining) amphiphysin like 2/32 (6%) CV2 like 1/32 (3%)	Hu like 10/14 (71%) amphiphysin like 4/14 (29%)	Hu like 2/3 (66%) Yo like 1/3 (33%)	Hu like 1/2 (50%) amphiphysin like 1/2 (50%)

## Discussion

This report demonstrates for the first time that a commercially available assay used in an NHS immunology laboratory can be reliably used to provide further evidence of a possible immune mediated pathogenesis in the context of PACA.

Using a commercial indirect immunofluorescent assay for the detection of well-characterised paraneoplastic antibodies offered by our NHS immunology laboratory, we have made the observation that sera from patients with suspected immune ataxias show positive results in 91% as opposed to 5 and 4% in patients with ataxia due to a genetic or a degenerative cause respectively. In none of the positive patients was immunoblot positive (we had excluded patients with PCD), in contrast to what is seen in those cases of PCD. This readily available commercial assay therefore provides a useful additional diagnostic aid for suspected IMCA. A positive result is particularly helpful in the context of PACA where the diagnosis relies on fulfilling recently published diagnostic criteria but without any specific single test being diagnostic.

**Fig. 1:**
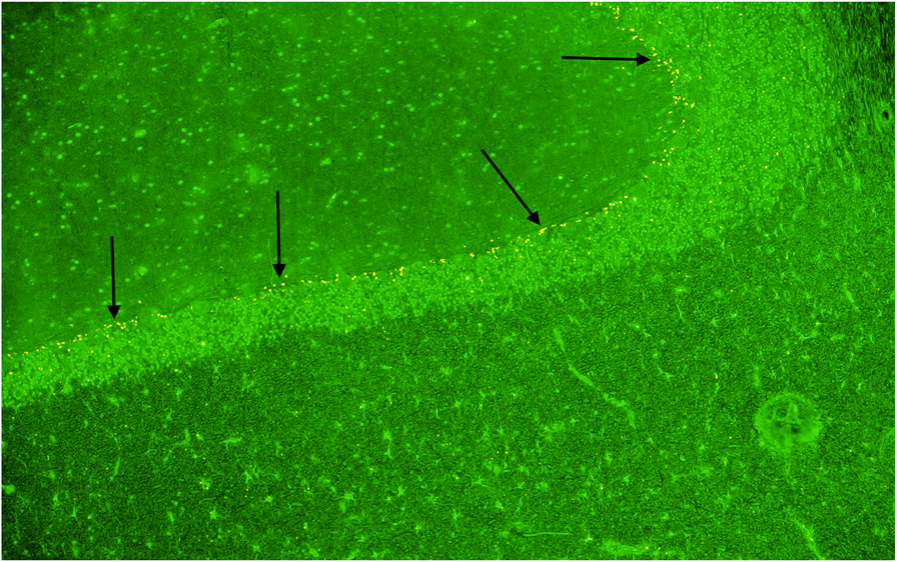
An example of a Hu like immunofluorescence staining (arrows) from a patient with suspected immune mediated ataxia. Immunoblot was negative for anti-Hu. This pattern of staining was the commonest observed in all the immune ataxia groups

The vast majority of positive results reported here showed an immunoreactivity mimicking what is seen in anti-Hu antibody related PCD (Fig. 1). Less common patterns seen included anti-Yo, anti-CV2, anti-amphyphysin and anti-Tr antibodies. However subsequent immunoblot was negative for any of these antibodies, eliminating the likelihood of PCD.

For patients with gluten ataxia and anti-GAD ataxia, specific diagnostic markers already exist in the form of antigliadin and/or TG6 antibodies and anti-GAD antibodies respectively. The fact that sera from patients with GA or anti-GAD ataxia demonstrate reactivity with cerebellar tissue also supports the fact that these ataxias are indeed immune-mediated. Whilst further work on characterising the specificity of antibody reactivity and pathogenicity has already been done in the context of GA and anti-GAD ataxia, this report highlights the utility of a simple immunofluorescence assay as a useful tool in raising the suspicion of immune mediated ataxias [7–10]. This is particularly helpful in the context of PACA. PACA is the term used to describe autoimmune ataxias where the antigenic trigger is not known and any associated neuronal antibodies are not well-characterized or of proven pathogenicity [3]. Whilst diagnostic criteria for PACA have recently been published, any additional serological tests such as what has been described here may be useful in increasing the diagnostic suspicion for PACA.

A previous smaller study by our group, used an in-house immunohistochemistry study and showed reactivity using rat cerebellum in 12/20 (60%) patients with idiopathic sporadic ataxia (not necessarily selected for the possibility of autoimmune pathogenesis) as opposed to 1/20 (5%) in patients with genetic ataxias [11].

The expanding spectrum of cerebellar, and in particular Purkinje cell antibodies, in the context of both PCD and other immune ataxias, has been highlighted in a series of review articles by Jarius and Wildemann in which they discus the clinical, oncological, therapeutic and pathogenic features of the 12 most common Purkinje cell antibodies [12]. Whilst very specialised immunological laboratories can offer such a service, accessibility to such testing is limited for most neurologists who encounter a patient with potentially immune mediated ataxia. Furthermore, by definition PACA is not associated with such pathogenic antibodies but is likely to represent a heterogeneous entity of immune-mediated ataxias for which, so far, no specific pathogenic antibodies have been described.

In summary we present our data based on a large cohort of patients with ataxia who were tested using a commercial immunofluorescence assay, demonstrating the presence of reactivity using monkey cerebellar tissue in those patients with IMCA. Such reactivity was rare in genetic and degenerative ataxias and as such this commercially available assay can be a useful and practical screening tool, particularly for patients suspected of having PACA where a single diagnostic marker does not exist.

## Data Availability

The corresponding author had full access to all the data and takes responsibility for the integrity of the data and the accuracy of the data analysis. Anonymised data can be made available upon request.
